# Disturbance History Shapes Microbial Community Assembly and Predictability in a Continuous Culture System

**DOI:** 10.1111/1462-2920.70430

**Published:** 2026-09-27

**Authors:** Sophia. E. Renes, Berenike Bick, Brendan Gerard McKie, Hannah B. Fried‐Petersen, Silke Langenheder, David G. Angeler, Eva S. Lindström

**Affiliations:** ^1^ Department of Aquatic Sciences and Assessment Swedish University of Agricultural Sciences Uppsala Sweden; ^2^ Department of Ecology and Genetics/Limnology Uppsala University Uppsala Sweden; ^3^ Centre for Biodiversity Dynamics (CBD), Department of Biology Norwegian University of Science and Technology (NTNU) Trondheim Norway; ^4^ Consejo Superior de Investigaciones Científicas, Museo Nacional de Ciencias Naturales (MNCN‐CSIC) Madrid Spain; ^5^ School of Natural Resources University of Nebraska ‐ Lincoln Lincoln Nebraska USA; ^6^ IMPACT, the Institute for Mental and Physical Health and Clinical Translation Deakin University Geelong Victoria Australia

## Abstract

Legacy effects that arise when past environmental conditions influence present community responses are increasingly recognized as important drivers of microbial dynamics. However, how repeated disturbances and the intervals between them shape community assembly, and whether they lead to predictable trajectories or more contingent outcomes, remains poorly understood. Here, we used a controlled continuous culture experiment to examine how two alternative histories of repeated minor salinity pulses shaped bacterial community responses to a subsequent major salinity pulse. Using a high‐resolution time series of 26 sampling points over 50 days, we detected systematic treatment effects and increasing divergence among within‐treatment replicates over time. Community responses to the second salinity pulse depended on disturbance history: Previous exposure altered both the magnitude and direction of richness change, and even promoted richness when recovery time was brief. Moreover, exposure to the subsequent salinity disturbance maintained elevated levels of inferred stochasticity in communities with disturbance histories following the major pulse. Together, these findings demonstrate that disturbance sequence and recovery interval critically shape microbial stability and influence the balance between stochastic and deterministic assembly processes.

## Graphical abstract

Outline of the experiment made where bacterial communities were exposed to different disturbance treatments, and the following community assembly was analysed. Figure (a) shows the setup of the experiment and Figure (b) shows the expected outcome in variation among treatment replicates over time.

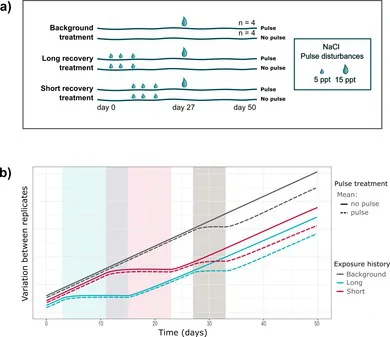

## 1 Introduction

Over decades of ecological research many processes involved in shaping ecological communities have been described. Vellend (2010, 2016) states that virtually all processes fit under one or more of the following four so‐called higher level processes: (1) Selection; (2) Dispersal; (3) Drift and (4) Speciation. These processes can be either stochastic, like drift, or deterministic, as selection or both, for example, speciation and dispersal (Chase and Myers 2011; Stegen et al. 2012; Vellend et al. 2014).

In microbial communities, there is strong support for the idea that deterministic processes are very important for community assembly (Martiny et al. 2006; Lindström and Langenheder 2012; Nemergut et al. 2013; Langenheder and Lindström 2019). For example, selection is a deterministic process where fitness differences between individuals from different species lead to changes in community composition in response to environmental filtering via abiotic factors and species interactions (Vellend 2010, 2016) and is commonly observed (e.g., Langenheder and Lindström 2019). On the contrary, stochastic processes were originally thought to be less important in microbial community assembly, but have gained increasing attention (Zhou and Ning 2017). It now acknowledged that deterministic and stochastic processes simultaneously can affect microbial community assembly (Santillan et al. 2025). For example, studies have shown that initially similar communities diverge over time under similar environmental conditions, indicating that stochasticity, including processes such as drift related to random birth and death events, can also play an important role (Zhou et al. 2013; Gundersen et al. 2021).

Environmental disturbances can reduce microbial diversity and the ecosystem processes they mediate (Steudel et al. 2012). Salinity, in particular, strongly influences microbial diversity and community composition (Song et al. 2022). This can be important in estuarine environments, where freshwater and seawater mix and salinity gradients fluctuate greatly (Lozupone and Knight 2007). However, the specific consequences of such disturbances for bacterial communities remain less well understood (Graham et al. 2016). Current evidence suggests that disturbed bacterial communities are often predominantly shaped by deterministic processes, driven by increased selection pressure, which can reduce local diversity (Wang et al. 2022; Chen et al. 2023; Odhiambo et al. 2023; Dai et al. 2023). Several experimental studies support this pattern (Gundersen et al. 2021; Santillan et al. 2019), and in some cases, bacterial communities may not fully recover from disturbances (Santillan et al. 2020). However, intermediate levels of disturbance can increase stochasticity in community assembly, resulting in less predictable outcomes, and also affect community functioning (Santillan et al. 2019; Santillan and Wuertz 2022).

The filtering effect of past disturbances on community composition might ‘protect’ communities from the effects of subsequent disturbances through stress‐induced community tolerance (Blanck 2002; Vinebrooke et al. 2003) or acquired stress resistance (Rillig et al. 2015; Andrade‐Linares et al. 2016), ultimately shaping the adaptive capacity of ecosystems (Angeler et al. 2019). Such legacies of past disturbances might also be transient, creating a ‘short memory’ (Cabrol et al. 2016; Vass and Langenheder 2017). ‘Memory’ should here be understood as a phenomenon including several different processes, such as adaptations and selection of different individuals possessing certain traits as a result of a past disturbance (Johnstone et al. 2016). In general, little is known about how time since last disturbance, as well as other properties of past disturbances, affect the ability of microbial communities to cope with subsequent disturbances (Philippot et al. 2021). More specifically, it is unclear when legacy effects increase the adaptive capacity or when they, on the contrary, erode it, leading to a persistent reorganization of community structure and function.

We aimed to determine how previous pulse disturbances, and the recovery time since the last disturbance, shape bacterial community responses to a new, stronger disturbance. Community responses were quantified as changes in alpha‐ and beta‐diversity. We hypothesized that these changes arising as a result of a disturbance would be reduced in communities with a history of previous exposure to the same type of disturbance. Moreover, we hypothesized that this protective memory effect of disturbance history would be transient and weaken with a longer time elapsed since last disturbance. To test our hypotheses, we cultivated a freshwater bacterioplankton community in continuous culture, creating different exposure histories of repeated minor salinity pulses followed by long and short recovery periods, and a subsequent major salinity pulse disturbance (Figure 1a, see methods for more details).

**FIGURE 1:**
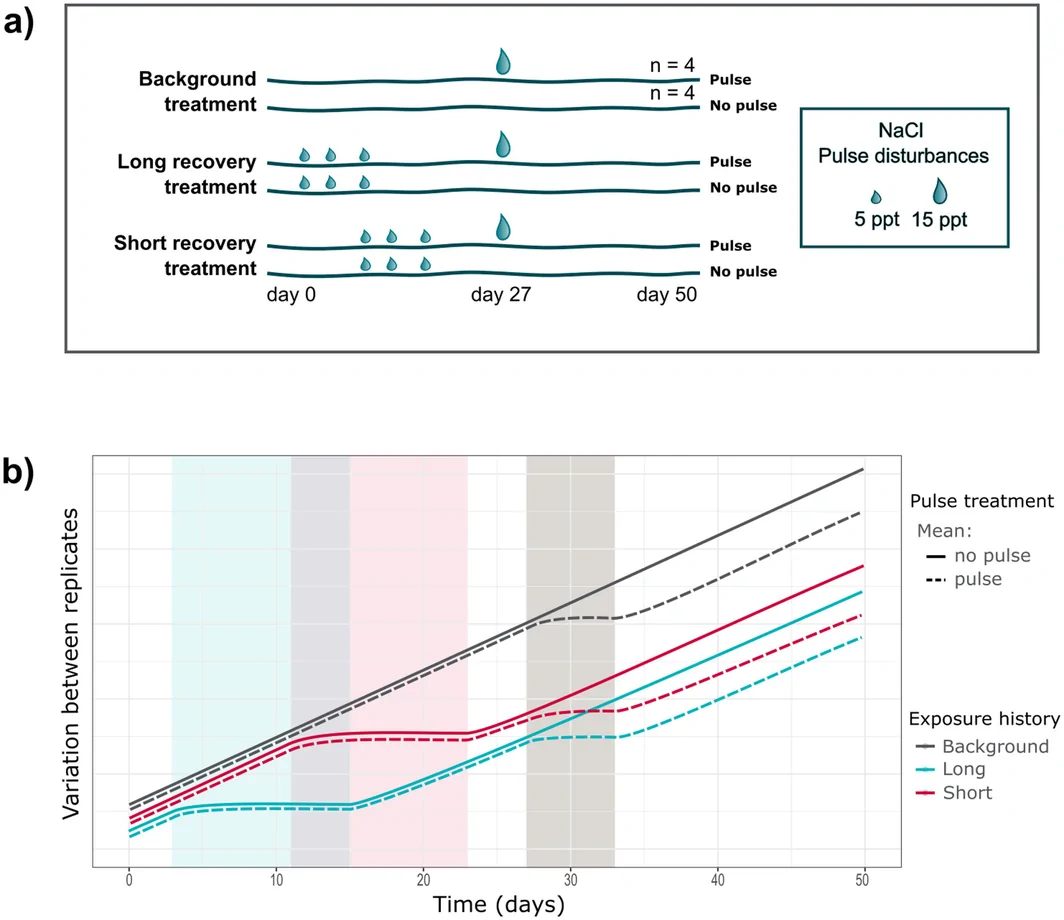
(a) Experimental set‐up showing the 3 × 2 treatment groups, three different disturbance histories (control, long and short recovery time) with each pulse and no pulse conditions. Each treatment was replicated four times. (b) Expected variation between replicates over time for the different treatments (shown in different colours and line types). Blue shaded area shows the timing of the minor salinity pulses for the long disturbance history and red for the short disturbance history, whereas the grey shaded area shows the timing of the major salinity pulse.

In addition to selection based on ‘memory’ effects, our experiment allowed us to evaluate the possible influence of stochasticity. The latter should manifest, given dispersal was limited, as variation among replicates that cannot be systematically attributed to the disturbance history treatments. Based on previous research (Evans et al. 2017) we expect that stochasticity in our experiment would be visible as (1) increased beta‐diversity among communities (across replicates within a treatment) over time, and (2) increased importance of stochasticity compared to determinism in community assembly as shown through null modelling. Further, we hypothesized that stochasticity, and, thus, divergence between the replicates, would be temporarily halted or reduced for the duration of the series of minor disturbances in the long and short recovery treatments and for the duration of the major pulse disturbance in all disturbance history treatments (Figure 1b). During the remainder of the study period, this divergence was expected to continue at a steady rate.

## 2 Material and Methods

### 2.1 Experimental Set‐Up

The experiment was performed with bacterioplankton communities grown in a continuous culture setup. There were 3 × 2 treatment groups, with four replicates each (24 vessels in total, Figure 1a). Three different exposure histories (eight vessels per disturbance history treatment) were created during the first 27 days of the experiment by administering a series of three minor salinity disturbances (5 ppt NaCl each) with 3 days between the 1st and 2nd minor pulse and 5 days between the 2nd and 3rd minor pulse, and varying recovery times. The ‘long recovery treatment’ and ‘short recovery treatment’ were followed by two‐ and one‐week recovery periods, respectively, after the last minor pulse, while the ‘background treatment’ served as the control for this factor and was left undisturbed during this period (Figure 1a). Half (4) of the replicates from each disturbance history treatment were then exposed to a single, stronger, salinity pulse (15 ppt NaCl). After this disturbance, the communities were allowed to grow in continuous culture for an additional 23 days under background laboratory conditions. Considering the origin of the bacterial community (see below), we did not expect any previous exposure to salinity, so that any salinity addition would likely be a disturbance. The strengths of the salinity pulses were chosen based on results from previous experiments conducted at our laboratory using freshwater bacteria (Berga et al. 2012), which indicated that salinity changes of c. 5 and > 10 PSU constitute intermediate and strong disturbances, respectively. The timing of the disturbances was chosen in relation to the generation time of the bacterial community in continuous culture, where we aimed to allow at least one generation to pass between each of the repeated minor disturbances, and to allow several generations to pass between the disturbance history and the major pulse. The goal, by doing this, was to focus on community level processes, rather than mainly physiological (within one generation) or evolutionary (over the course of many generations) processes.

Since the salt was gradually diluted out of the vessels due to the continuous culture setup, it is not straightforward to determine exactly when the disturbances ended. However, we estimate that the disturbances ended at the latest 4 days after the last minor salinity pulse and 6 days after the major pulse, as that is when the conductivity levels returned to below 1500 μS/cm, which is the high end of the conductivity range associated with freshwater (data not shown). Note that samples for analysis of the bacterial communities were always taken before disturbances were applied.

### 2.2 Source Materials and Laboratory Conditions

The bacterial community used to initiate the experimental communities was collected in a sterile bottle from a freshwater forest stream in the Uppland region of Sweden (59°55′05.0″ N, 17°20′49.2″ E). The site was upstream of Lake Fiby (conductivity: 9.60 mS m^−1^; pH: 7.30; alkalinity/acidity: 0.516 mekv L^−1^; total organic carbon: 27.9 mg L^−1^ C; total nitrogen: 1070 μg L^−1^ N; total phosphorus: 37.9 μg L^−1^ P; (Miljödata‐MVM 2020)). The microbial community was filtered through 0.7 μm filters (Whatman GF/F grade filters), to remove potential predatory organisms, and was then allowed to acclimatize at laboratory conditions. The acclimatization consisted of 4 days in batch culture, without added medium, followed by 3 weeks in continuous culture under the same conditions as during the main experiment. Each culture vessel contained 200 mL of the bacterial community growing in medium. We used an artificial lake water medium, made according to Bastviken et al. (2004). As a carbon source, we used 10 mg L^−1^ autoclaved dissolved organic carbon collected from the same stream used to source the starting communities using a reverse osmosis machine (Realsoft, Norcross, GA, USA). Medium was pumped through the vessels at a continuous rate of 70.4 μL min^−1^ throughout the experiment, which equals a dilution rate of 0.02 h^−1^ and a generation time of 32.8 h. The dilution rate is the medium flow rate divided by the culture volume and the generation time is ln(2) divided by the dilution rate (Ziv et al. 2013). In order to counteract any divergence between the communities that might have occurred during acclimation, we homogenized the communities from the 24 vessels and redistributed them before starting the experiment. The experiment took place at 20°C and in the dark. The communities were stirred using magnetic stirrers to prevent flocculation and formation of biofilms in the vessels (Ziv et al. 2013).

### 2.3 Sampling and Administration of Disturbances

Every second day 20 mL samples were taken from all replicates for analysis. The samples were taken from the water column through a mounted syringe connected to a three‐way stop‐cock valve to avoid contamination while regulating access to the vessels. A solution of 350 g L^−1^ NaCl was prepared and autoclaved before adding 2.86 or 11.44 mL to each vessel to reach the intended disturbance intensities of 5 ppt and 15 ppt NaCl, respectively.

To control for possible effects of the physical disturbance associated with addition of the salt solution, all replicates not receiving a salinity pulse at any given disturbance point instead received autoclaved Milli‐Q water identical to the volume of the salt solution added to the disturbed communities. All salinity pulse disturbances and control treatments were administered using the mounted syringe described above. Each vessel had an air tube with a 0.2 μm air filter to prevent pressure buildup in the system.

### 2.4 Analysis of Bacterial Community Composition

For community composition analysis, 10 mL of culture from each community was filtered onto a 0.2 μm filter (PALL) to collect the bacterial cells. The filters were stored at −80°C while awaiting processing. DNA was extracted from the filters using DNeasy Powersoil DNA extraction kits (Qiagen, Maryland, USA) and stored at −20°C until further processing. The 16S rRNA genes (V3 and V4 regions) were amplified by polymerase chain reaction (PCR) in two steps (see Methods S1 for detailed information). Sequencing was performed by the SNP&SEQ Technology Platform (Illumina MiSeq, v.3 sequencing chemistry) in Uppsala. Demultiplexed sequence data from the 624 samples collected during the experiment were processed using the DADA2 pipeline (DADA2 R package version 1.8, using R version 3.4.3, (Callahan et al. 2016)). The sequences were truncated after 290 nucleotides for forward and 240 for reverse reads. Amplicon sequence variants (ASVs) were identified and chimeras were removed after merging read pairs, leading to 16% chimera removal. Taxonomy was assigned to the ASVs using the SILVA SSU 132 reference database.

### 2.5 Statistical Analysis of Community Data

Samples with fewer than 10,000 reads (23 samples out of 624) were excluded from further analysis. This resulted in 1304 ASV's and an average of 23,753 reads per sample. All analyses were performed in R (version 4.4.2). To account for the compositional nature of the ASV data, we followed the framework proposed by Gloor et al. 2017. For ASV richness, sequencing depth was standardized across samples by randomly subsampling ASVs to 10,245 reads per sample using *rrarefy* from the vegan package (version 2.6‐8, Figure S1). This rarefaction step was performed to minimize biases arising from unequal sequencing depth among samples and to enable comparable diversity estimates. To quantify temporal changes in ASV richness after the major pulse disturbance across the different treatments (control, short and long recovery history), we used log response ratios (LRR; Hedges et al. 1999). For each treatment (control, long and short recovery history), we computed:

$$\mathrm{LRR}=\log\left(\frac{\text{speciess richness in major pulse}}{\text{mean species richness in}\;\mathrm{no}\;\text{major pulse}}\right)$$

where values close to zero indicate similarity to no major pulse conditions, and larger positive or negative values indicate stronger deviation from no major pulse conditions. We focused the LRR analysis on ASV richness because richness directly reflects ASV turnover following disturbance and provides an interpretable measure of community‐level change. To account for natural variability among replicates, LRRs were also calculated among no‐major‐pulse replicates.

To visualize overall treatment effects on community composition, we performed a principal coordinate analysis (PCoA) with Aitchison distances, the Euclidean distance over centred log‐ratio (CLR) transformed data, on the global ASV data. This approach is appropriate for compositional microbiome data because it accounts for the relative nature of sequence counts. To test the effects of disturbance history, major‐pulse treatment, time and their interactions on community structure, we performed factorial PERMANOVA on Aitchison distance matrices using adonis2 in the vegan package with 999 permutations. Analyses were conducted across the complete experiment and separately for the periods before (Days 1–27) and after (Days 28–50) the major pulse. Time was included as a continuous predictor, and model terms were evaluated sequentially (by = ‘terms’). Homogeneity of multivariate dispersion was assessed for disturbance history, major‐pulse treatment where applicable, and sampling time using PERMDISP (betadisper) on the corresponding Aitchison distance matrices. Distances to group centroids were calculated, and significance was evaluated using 999 permutations. To evaluate differences between replicates per treatment and time point, we used pairwise Aitchison distance on the original abundances using *robust aitchison* implemented in *vegdist* in the vegan package (Martino et al. 2019). This approach first replaces zeros with a small pseudocount, equal to half of the minimum nonzero relative abundance, and then applies CLR transformation. Euclidean distances are then computed in this CLR space. This approach quantifies proportional differences in community composition and is appropriate for compositional data (Aitchison 1982; Aitchison et al. 2000).

To assess the relative roles of stochastic versus deterministic processes in community assembly, we performed null model analysis using the normalized stochasticity ratio (NST; Ning et al. 2019). NST compares the observed community dissimilarity, abundance‐weighted Aitchison distances computed with the robust Aitchison method, to a distribution of dissimilarities generated under a specified null model. We used *tNST* in package NST (version 3.1.10) with a Proportional‐Fixed (PF) null model and 999 randomizations. NST values range from 0 to 1 and reflect the relative balance between deterministic and stochastic assembly processes, with lower values indicating a stronger relative contribution of deterministic processes and higher values indicating a stronger relative contribution of stochastic processes.

Linear mixed‐effects models (LMM) were used to evaluate how disturbance history (i.e., control, long recovery and short recovery treatments) and the major salinity pulse affected each of our response variables (Table S1). We conducted two sets of LMMs, the first focused on samples conducted prior to the major salinity pulse (i.e., with only samples collected before Day 27 were included) and a second focused on samples collected after the major salinity pulse. ASV richness prior to the major pulse was modelled as a function of disturbance history, sampling day and their interaction as fixed effects, with replicate identity included as a random intercept. Prior to the major salinity pulse, each disturbance‐history treatment consisted of *n* = 8 replicates sampled repeatedly through time. Because the major pulse had not yet been applied, replicates that were later assigned to the major‐pulse and no‐major‐pulse treatments were analysed together within each disturbance‐history treatment.

For LMMs of samples collected after the major salinity pulse on Day 27, the analysis included *n* = 4 replicate per treatment, with ASV richness analysed using log‐response ratios (LRR), calculated as the difference between major‐pulse and no‐major‐pulse treatments within each disturbance‐history treatment. LRRs were modelled as a function of disturbance history, with sampling day included as a random intercept. To assess treatment effects on pairwise Aitchison distances and normalized stochasticity ratios (NST) before and after the major pulse, linear mixed‐effects models included disturbance history, centred sampling day, major pulse treatment (when applicable) and their interactions as fixed effects. Pairwise comparison identity or replicate identity was included as a random effect, depending on the response variable, to account for repeated sampling through time. Continuous predictors were centred where appropriate to aid interpretation. Degrees of freedom and *p*‐values were estimated using Satterthwaite's approximation implemented in the lmerTest package. Model residuals were visually inspected to confirm assumptions of homoscedasticity and normality. Because analyses were hypothesis‐driven and response variables represented distinct ecological properties, *p*‐values were not adjusted for multiple comparisons.

## 3 Results

### 3.1 ASV Richness

ASV richness declined across all treatments during the early phase of the experiment, reaching a minimum prior to the major pulse on Day 27 (Figure S2). Prior to the major salinity pulse (Day 27), disturbance history, time and their interaction significantly affected ASV richness (Table 1). ASV richness declined over time in all treatments during the early phase of the experiment (Figure S2). After the major pulse on Day 27, richness remained relatively stable in the control and long recovery treatments, whereas stronger temporal fluctuations were observed in the short recovery treatment. Overall, post‐pulse richness remained below initial levels across treatments (Figure S2). To assess the effect of the major pulse across disturbance histories, we used log response ratio (LRR) of ASV richness for each recovery history (control, long and short recovery; Figure 2) comparing communities receiving a major pulse with those that did not. Both the long and short recovery treatments showed significantly higher LRR values compared to the undisturbed control (Table 2). Thus, communities with a history of disturbance increased in richness after the major pulse with the short recovery treatment showing the strongest positive response (Figure 2). In contrast, communities lacking disturbance history (i.e., controls) decreased in richness if receiving the major pulse.

**TABLE 1:** Results of linear mixed‐effects models testing the effects of disturbance history (control, long recovery, short recovery), time (day), and their interaction on ASV richness prior to the major salinity pulse (Day 27).

Response	Effect	df (Num, Den)	*F*‐value	*p*
ASV richness	Disturbance history	2, 311.03	6.58	**0.0016**
ASV richness	Time	1, 311.04	87.83	**< 0.001**
ASV richness	Disturbance history × Time	2, 311.01	3.37	**0.0358**

*Note:* Only samples collected before Day 27 were included in the analysis. Replicate identity was included as a random effect. Significant (*p* < 0.05) values are indicated in bold.

**FIGURE 2:**
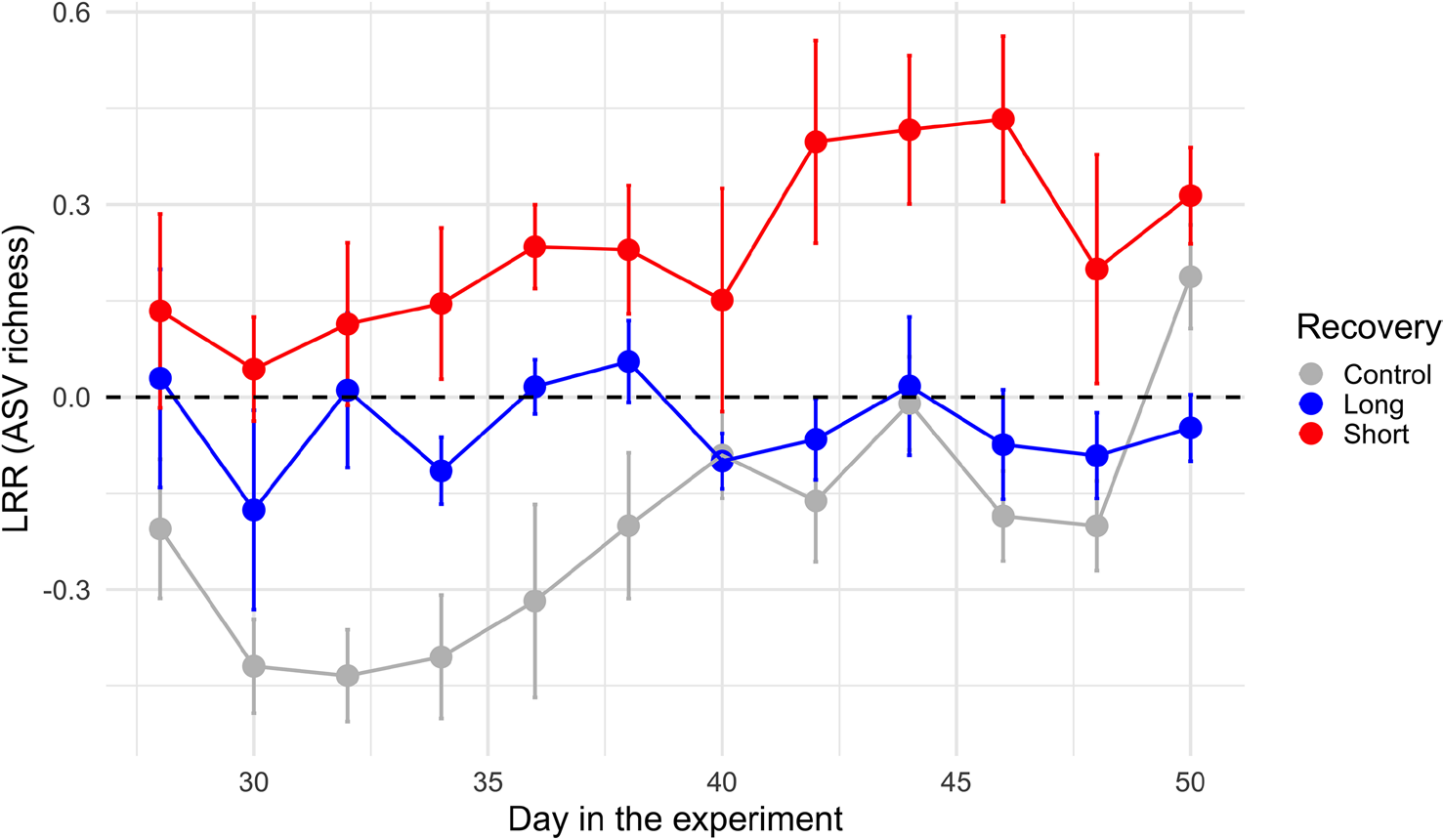
Log response ratios (difference between major pulse and no major pulse conditions for each disturbance history: grey = control, blue = long and red = short recovery) for ASV richness over time after the major salinity pulse on Day 27. Points show means and error bars standard errors.

**TABLE 2:** Results of linear mixed‐effects models testing the effects of disturbance history, major pulse treatment, time and their interactions on community assembly metrics before and after the major salinity pulse (Day 27).

Response	Effect	Estimate	SE	df	*t*‐value	*p*
Before pulse (Day 1:27)						
Pairwise Aitchison distance	(Intercept)	19.57	0.10	1070.00	193.93	**0.000**
	Disturbance history (long)	0.93	0.14	1070.00	6.46	**< 0.001**
	Disturbance history (short)	0.59	0.14	1070.00	4.10	**< 0.001**
	Time (centred)	0.14	0.01	1070.00	11.12	**< 0.001**
	Disturbance history (long) × Time (centred)	−0.01	0.02	1070.00	−0.31	0.755
	Disturbance history (short) × Time (centred)	−0.03	0.02	1070.00	−1.62	0.105
NST	(Intercept)	0.60	0.01	156.25	86.74	**< 0.001**
	Disturbance history (long)	0.09	0.01	1044.28	9.53	**< 0.001**
	Disturbance history (short)	0.02	0.01	1044.29	2.49	**0.013**
	Time (centred)	0.01	0.00	1048.40	15.17	**< 0.001**
	Disturbance history (long) × Time (centred)	0.00	0.00	1044.13	−0.93	0.352
	Disturbance history (short) × Time (centred)	0.00	0.00	1045.58	−0.86	0.390
After pulse (Day 28:50)						
LRR	(Intercept)	−0.19	0.04	27.40	−4.92	**< 0.001**
	Disturbance history (long)	0.16	0.05	22.00	3.52	**0.002**
	Disturbance history (short)	0.45	0.05	22.00	9.89	**< 0.001**
Pairwise Aitchison distance	(Intercept)	21.77	0.25	92.49	88.26	**< 0.001**
	Disturbance history (long)	−1.82	0.32	395.02	−5.62	**< 0.001**
	Disturbance history (short)	−2.56	0.32	395.05	−7.91	**< 0.001**
	Major pulse	−0.95	0.32	395.62	−2.95	**0.003**
	Time (centred)	−0.03	0.04	394.88	−0.78	0.436
	Disturbance history (long) × Major pulse	2.18	0.44	394.59	4.91	**< 0.001**
	Disturbance history (short) × Major pulse	3.70	0.45	394.92	8.29	**< 0.001**
	Disturbance history (long) × Time (centred)	−0.02	0.05	394.95	−0.46	0.648
	Disturbance history (short) × Time (centred)	0.01	0.05	394.48	0.25	0.801
	Major pulse × Time (centred)	0.06	0.05	394.53	1.32	0.188
	Disturbance history (long) × Major pulse × Time (centred)	−0.10	0.06	394.56	−1.61	0.107
	Disturbance history (short) × Major pulse × Time (centred)	−0.01	0.07	394.61	−0.22	0.829
NST	(Intercept)	0.74	0.02	24.13	35.88	**< 0.001**
	Disturbance history (long)	−0.09	0.02	394.30	−3.98	**< 0.001**
	Disturbance history (short)	−0.11	0.02	394.31	−5.02	**< 0.001**
	Major pulse	0.04	0.02	394.53	1.67	0.096
	Time (centred)	0.00	0.00	394.25	0.38	0.706
	Disturbance history (long) × Major pulse	0.08	0.03	394.15	2.73	**0.007**
	Disturbance history (short) × Major pulse	0.05	0.03	394.27	1.48	0.140

	Disturbance history (long) × Time (centred)	−0.01	0.00	394.28	−1.81	0.070
	Disturbance history (short) × Time (centred)	0.00	0.00	394.11	−0.31	0.754
	Major pulse × Time (centred)	0.00	0.00	394.13	−0.11	0.909
	Disturbance history (long) × Major pulse x Time (centred)	0.00	0.00	394.14	−0.03	0.975
Disturbance history (short) × Major pulse x Time (centred)	0.00	0.00	394.15	−0.04	0.969

*Note:* Prior to the major pulse, analyses included only samples collected before Day 27 and tested the effects of disturbance history (control, long recovery, short recovery), time and their interaction. Following Day 27, models additionally included major pulse treatment (major pulse vs. no major pulse). Replicate identity was included as a random effect to account for repeated sampling through time. Each treatment combination consisted of *n* = 8 replicates before and *n* = 4 replicates after the major pulse. Degrees of freedom and *p*‐values were estimated using Satterthwaite's approximation. Significant (*p* < 0.05) values are indicated in bold.

### 3.2 Beta‐Diversity

PERMANOVA revealed that disturbance history, major pulse, time and all their interaction significantly affected community composition (Table S2). PERMDISP showed that, across all time points, multivariate dispersion differed significantly among disturbance‐history treatments, between major‐pulse treatments and among sampling times (Table S3). Additionally, PCoA ordination indicates that time was a major factor structuring community composition with an increase in beta‐diversity over time (Figure 3, Figure S4a–c). Variation among replicate communities was also evident in their taxonomic composition at the final sampling day. Relative abundances at the phylum level showed distinct compositional differences among replicates within each disturbance‐history treatment (Figure S3).

**FIGURE 3:**
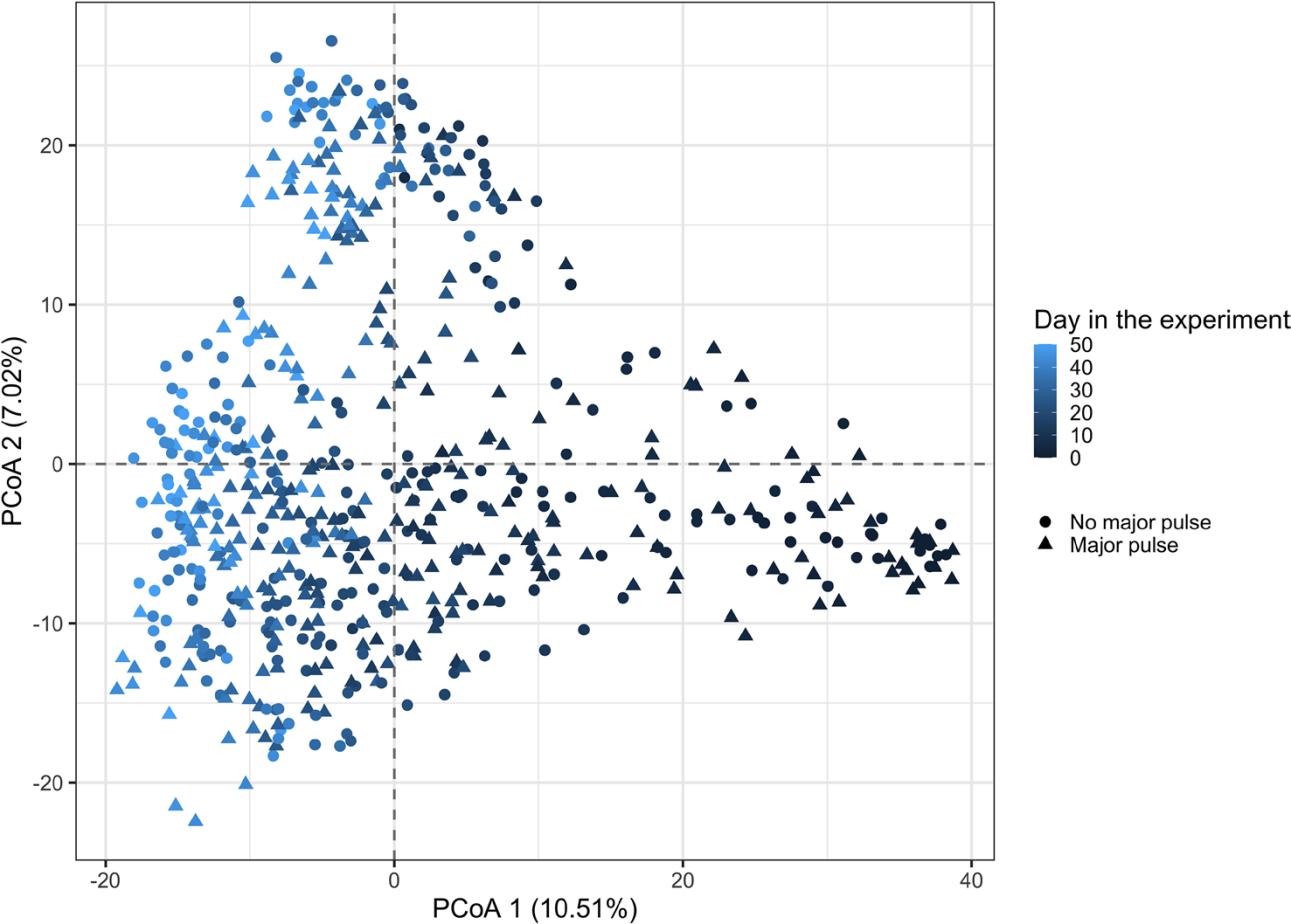
Principal coordinate analysis (PCoA) showing differences in bacterial community composition (based on Aitchison distances of 16S rRNA gene amplicon sequencing data) for no major pulse compared to major pulse treatments. The colour gradient indicates time (dark shades indicate early and lighter shades later days in the experiment).

We calculated pairwise Aitchison distances within each treatment and time point to quantify the divergence between replicates. Before the major pulse, pairwise Aitchison distances increased over time and slopes increased significantly more in both the long and short recovery treatments compared to the undisturbed control (Figure 4; Table 2), although the magnitude of these differences was relatively small. After the major pulse, Aitchison distances became flatter across treatments. There was a significant effect of disturbance history and the major pulse on Aitchison distance, as well as the interaction between the major pulse and disturbance history (Table 2). More specifically, in treatments with a disturbance history (both long and short recovery) Aitchison distances were higher in treatments receiving the major pulse compared to those not receiving it, while no such effect was visible in treatments without a disturbance history (Figure 4).

**FIGURE 4:**
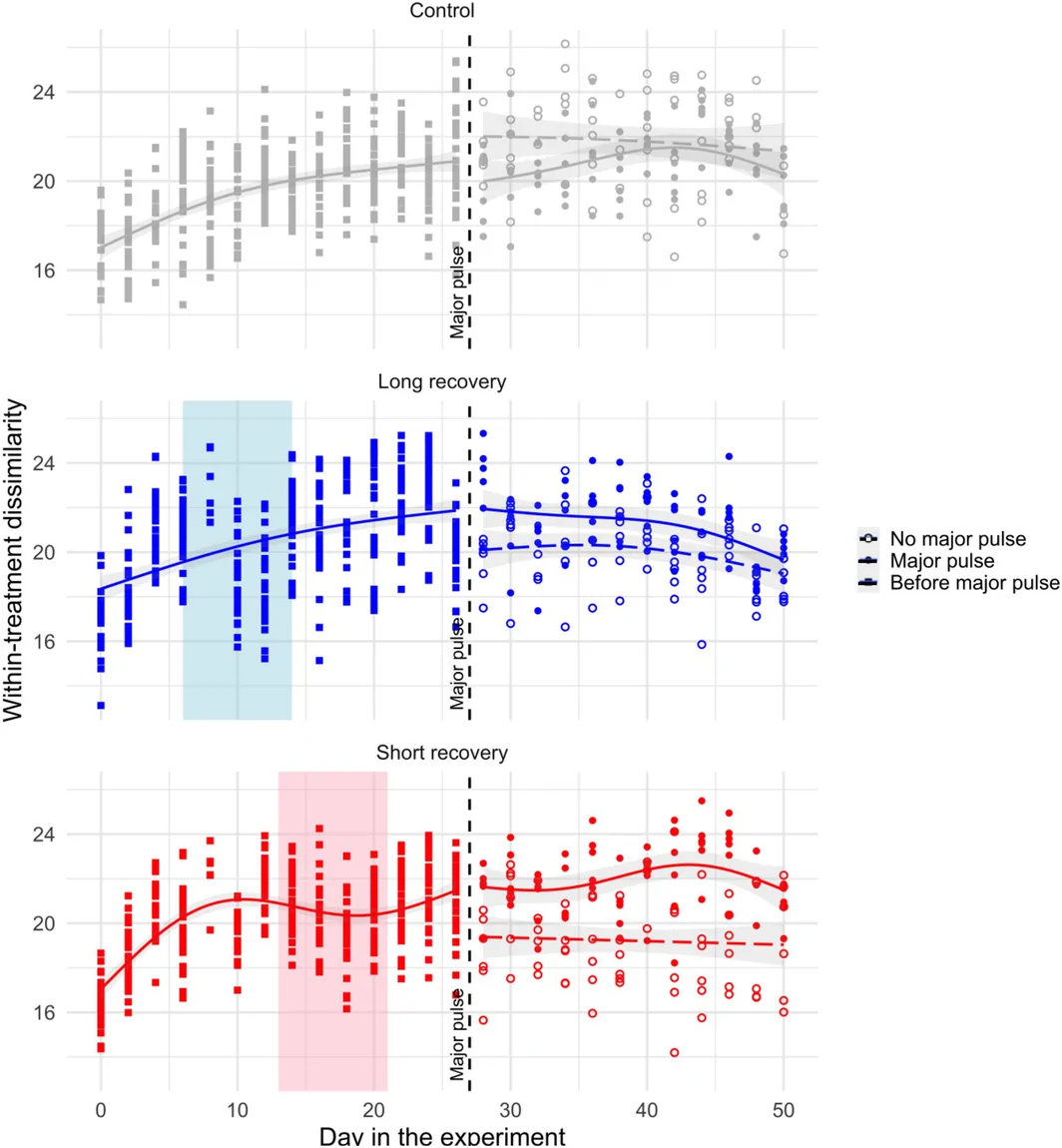
Pairwise Aitchison distance (dissimilarity between replicates) over time for each treatment: control (no disturbance history), long recovery treatment and short recovery treatment. Squares indicate samples collected before the major pulse on Day 27. Following the major pulse, filled circles represent treatments exposed to the pulse, whereas open circles represent treatments not exposed to the pulse. Solid and dashed lines represent GAM smoothers fitted to temporal trajectories for major pulse and no‐major pulse treatments, respectively; shaded areas indicate 95% confidence intervals. Blue and red shaded areas indicate the timing of the minor salinity pulses in the long and short recovery treatments, respectively.

### 3.3 Null Modelling

Results from the null model analysis showed that NST values exceeded 0.5 across all treatments and at nearly all time points (Figure 5). Prior to the major pulse, NST increased significantly over time, and both disturbance history treatments exhibited higher NST values than the undisturbed control (Table 2). Following the major pulse, disturbance history remained associated with differences in NST (Table 2; Figure 5), and the pulse itself had a close to significant effect (*p* = 0.094). The interaction between disturbance history and the major pulse was significant only for the long recovery treatment, in which communities exposed to the major pulse displayed consistently higher NST values than those that did not receive the pulse (Figure 5; Table 2).

**FIGURE 5:**
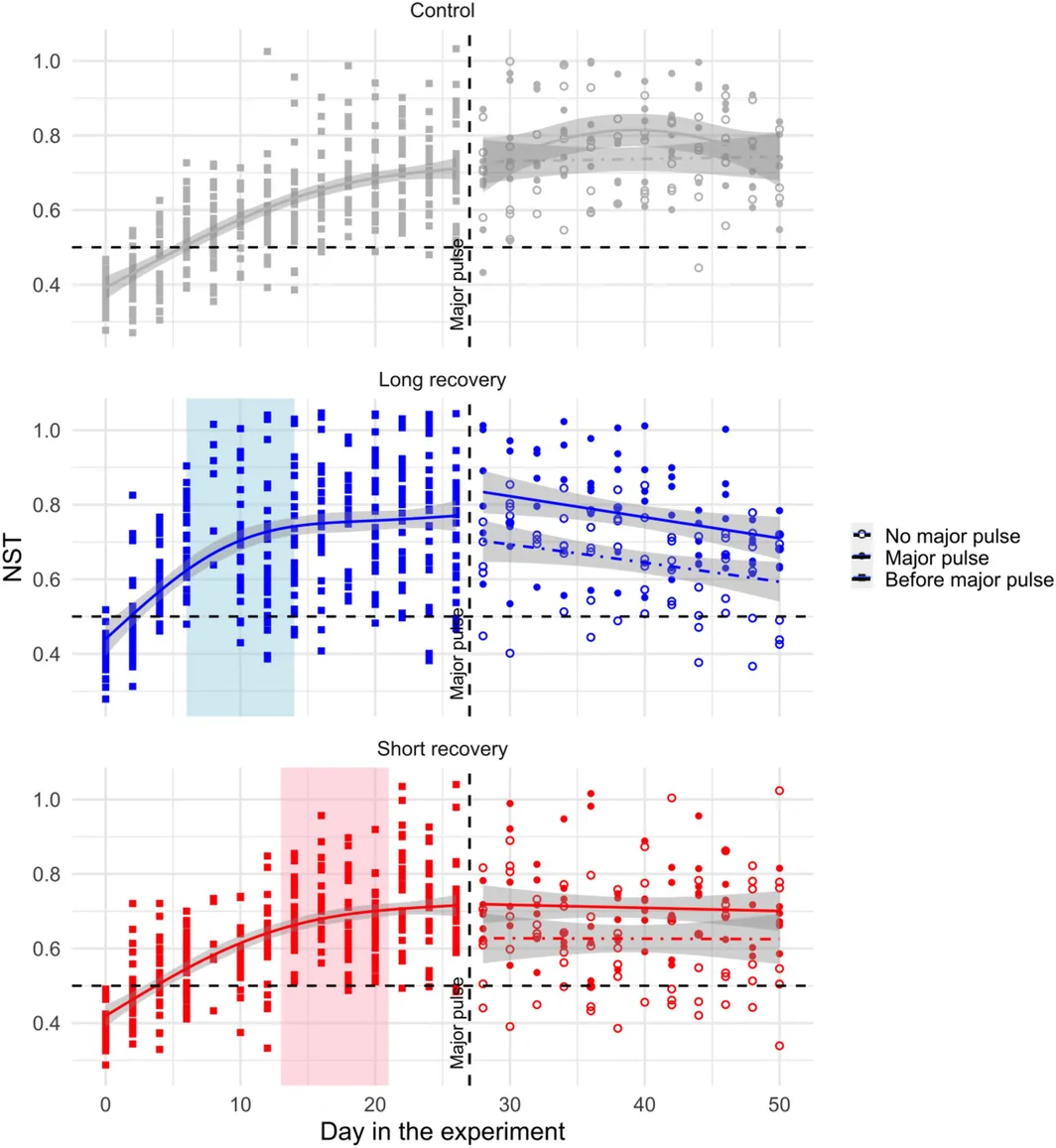
Normalized stochasticity ratio (NST; based on null modelling of pairwise Aitchison distances) over time for each treatment: control (no disturbance history), long recovery treatment and short recovery treatment. Squares indicate samples collected before the major pulse on Day 27. Following the major pulse, filled circles represent treatments exposed to the pulse, whereas open circles represent treatments not exposed to the pulse. Solid and dashed lines represent GAM smoothers fitted to temporal trajectories for major pulse and no‐major pulse treatments, respectively; shaded areas indicate 95% confidence intervals. Blue and red shaded areas indicate the timing of the minor salinity pulses in the long and short recovery treatments, respectively.

## 4 Discussion

We conducted a continuous‐culture experiment in which bacterial communities were exposed to repeated minor salinity pulses, followed, after different time lags, by a final major salinity pulse. We hypothesized that prior disturbance exposure would reduce microbial community sensitivity to later disturbances of the same type, and that the strength of this effect would diminish with increasing time between the previous and subsequent disturbance. Furthermore, we expected dissimilarity among replicate communities, and thus inferred stochasticity, to increase over time, but at a reduced rate during disturbance events.

Our study demonstrates that both the history and timing of prior disturbances shape bacterial community responses to a subsequent salinity pulse. In communities without a disturbance history (controls), the major salinity pulse disturbance decreased richness relative to communities that were not exposed to the pulse (Figure 2). Furthermore, richness in communities with a disturbance history (long and in particular short recovery) increased after the major pulse and even exceeded maximum richness of the control treatment. This result shows that legacy effects can buffer microbial communities against additional stress (Halbrook et al. 2024), perhaps because frequent disturbances before the major pulse selected for stress‐tolerant or opportunistic taxa, enabling resistance to further species loss (Jurburg et al. 2017; Peng et al. 2024; Oram et al. 2025). Although resistance and recovery were not directly quantified, this pattern suggests that frequent disturbances shortly before the major pulse preconditioned the community into a state that experienced reduced richness loss during the pulse and even an increase in richness afterwards (Allison and Martiny 2008; Shade et al. 2012; Zhou et al. 2014; Hawkes and Keitt 2015; Figure 2, Table 2). More generally, these findings are consistent with the concept of ecological memory, whereby past disturbances leave persistent imprints on community structure that shape subsequent responses to environmental change (Johnstone et al. 2016).

Overall beta‐diversity (Aitchison distance) was also affected by the disturbance treatments, and the effect of the major pulse over time varied with disturbance history, as hypothesized (Table S2). However, because multivariate dispersion differed among treatments, these PERMANOVA effects may reflect differences in group centroids, dispersion, or both (Table S3). Moreover, dissimilarity among replicate communities within a treatment increased over time and null model analyses showed that this dissimilarity was associated with increased inferred stochasticity in community assembly (Figure 5), indicating a strong influence of stochasticity under dispersal limitation, which is in line with previous research (Chase 2007; Zhou et al. 2014; Vellend et al. 2014; Le Moigne et al. 2023; Figures 3 and 4). Increased divergence among replicate communities indicates reduced predictability of community trajectories under identical environmental conditions. Contrary to our expectation (Figure 1b), divergence among replicates was not temporarily halted during the series of minor pulses. Before the major pulse, pairwise Aitchison distances increased more steeply in both the long and short recovery treatments than in the undisturbed control (Figure 4; Table 2), so the minor pulses accelerated rather than constrained divergence. This is consistent with the null model results discussed below, and suggests that repeated minor disturbances weakened deterministic filtering instead of imposing a common selective trajectory on replicate communities.

We expected selection to dominate under disturbance and stochasticity to become more influential under stable conditions, in the absence of disturbance (Vellend 2010; Dini‐Andreote et al. 2015; Zhou and Ning 2017; Albright et al. 2019). After the major pulse dissimilarity between replicates depended on disturbance history (Table 2). Most importantly, however, in both treatments with a disturbance history (long and short recovery), dissimilarity among replicates was higher in treatments receiving the major pulse, which was not the case in treatments without a disturbance history (Figure 4). Further, NST values were also higher in the long recovery treatment receiving the major pulse (Figure 5, Table 2). NST values should be interpreted comparatively within the context of this experimental system rather than as absolute indicators of deterministic versus stochastic assembly, because null model outcomes depend on the structure of the input data and model assumptions. The short recovery treatment was not associated with any significant difference between pulse treatments, though the trend was similar. Altogether, these results suggest that a history of disturbances can decrease the role of determinism caused by a later disturbance in community assembly, and, thereby, increase the relative contribution of stochasticity. Repeated minor salinity pulses may have pre‐filtered the community, which might have reduced fitness differences at the time of the major pulse and weakened selection, thereby increasing the relative influence of stochastic processes (Vellend 2010; Chase and Myers 2011; Nemergut et al. 2013). Repeated minor disturbances may also have selectively removed highly sensitive taxa prior to the major pulse, leaving communities composed of taxa with more similar stress tolerances and competitive capacities. Under such conditions, fitness differences during the major pulse may become reduced, weakening deterministic environmental filtering and increasing the relative influence of stochastic processes such as drift and priority effects.

Although, as reported above, a number of studies have used microbial communities to investigate the role of disturbances for stochasticity and the role of repeated disturbances for community resistance, resilience and composition, few have investigated the role of repeated disturbances for stochasticity, as we did here. Exceptions are the studies by Santillan et al. (2019) and Santillan and Wuertz (2022), who exposed activated sludge communities to repeated disturbances of different frequency and found the strongest role of stochasticity for community assembly at intermediate disturbance frequency when also the highest alpha‐diversity was observed. These results bear similarities to our findings where the communities with a disturbance history showed the highest alpha‐diversity as well as stochastic assembly following the major disturbance. Future work should investigate how different combinations of disturbance frequency, intensity and recovery duration interact to shape the balance between deterministic and stochastic assembly across microbial systems. More broadly, our results suggest that microbial responses to disturbance cannot be fully understood from disturbance intensity alone, but depend critically on the sequence and timing of prior environmental exposures. When interpreting these findings, it is important to acknowledge potential methodological limitations. The large number of samples (*n* = 624) constrained the achievable sequencing depth per sample. Alpha‐diversity measures are strongly dependent on sequencing depth. However, it has been shown that robust trends in alpha‐diversity among samples can be obtained at lower sequencing depths than applied here (Lundin et al. 2012). Further, rarefaction curves indicated that our sequencing depth captured the majority of the common taxa in each sample (Figure S1). However, detection of very rare taxa could have been limited, which can influence metrics sensitive to low‐abundance features, such as Aitchison distances and null model approaches. Increases in richness could also be understood as changes in the number of ASVs being above the detection limit rather than true changes in richness considering that the experiment was not open to recruitment by immigration, being performed in a closed system.

In our experiment, microbial communities were subject to dispersal limitation. If we had left replicates open to new dispersal during the experiment, we hypothesize that the importance of drift might have been smaller (Evans et al. 2017; Fodelianakis et al. 2021; Le Moigne et al. 2023). In natural systems, immigration, spatial heterogeneity and trophic interactions may modify these dynamics, potentially dampening or amplifying the role of stochastic assembly observed here. Future studies could, thus, include the effects of dispersal in experiments addressing the roles of disturbance and disturbance history because these factors combined may influence metaecosystem resilience (Angeler et al. 2023). However, on the other hand, the usefulness of both open and closed systems in experiments of this kind, for example for applications in biotechnology, has also been stressed (Santillan et al. 2025).

## 5 Conclusion

In summary, the unique time series of community samples in this study allowed us to closely follow community change in response to a series of salinity disturbances and a major disturbance event. Together, our findings demonstrate that bacterial community responses to disturbance are not only determined by the immediate stress but also by their disturbance history and recovery dynamics. Recent disturbances generate a short‐term ecological memory that enhanced richness, whereas longer intervals weakened this buffering effect. Additionally, a subsequent pulse increased inferred stochasticity in treatments with disturbance histories.

## Author Contributions

**Sophia. E. Renes:** conceptualization, investigation, writing – original draft, methodology, validation, visualization, writing – review and editing, formal analysis, supervision, data curation. **Berenike Bick:** writing – review and editing, writing – original draft, formal analysis, data curation, visualization. **Brendan Gerard McKie:** conceptualization, writing – original draft, writing – review and editing, project administration, supervision. **Hannah B. Fried‐Petersen:** writing – original draft, writing – review and editing, formal analysis, investigation. **Silke Langenheder:** conceptualization, writing – original draft, writing – review and editing, methodology, supervision, resources, funding acquisition. **David G. Angeler:** conceptualization, funding acquisition, writing – original draft, writing – review and editing, project administration, supervision. **Eva S. Lindström:** conceptualization, writing – original draft, writing – review and editing, project administration, supervision, resources, methodology.

## Funding

This work was supported by the Vetenskapsrådet (2014‐05828 and 2019‐03970), Knut och Alice Wallenbergs Stiftelse and National Genomics Infrastructure.

## Conflicts of Interest

The authors declare no conflicts of interest.

## Supporting information


**Figure S1:** Rarefaction curves of all samples (*n* = 601), the red line shows the read number to which the samples were rarefied (10,245 reads).
**Figure S2:** ASV richness over time for the different disturbance‐history treatments (grey = control, blue = long recovery, red = short recovery). Squares indicate samples collected before the major pulse on Day 27. Following the major pulse, filled circles represent major‐pulse treatments and open circles represent no‐major‐pulse treatments. Blue and red shaded areas indicate the timing of the minor salinity pulses in the long and short recovery treatments, respectively.
**Figure S3:** Relative abundances at phylum level in each replicate microcosm on the last day of the experiment (Day 50) represented with bars, top plots who no disturbance history, middle long and bottom short recovery history, no major pulse treatments are presented on the left and major pulse treatments on the right side, colours indicate different phyla.
**Figure S4:** PCoA for (a) before and (b) and (c) after the major pulse. The colour gradient indicates the days in the experiment and the symbols in (a) and (b) the different treatments (control, short and long recovery history) and in (c) if the treatments did or did not receive the major pulse.
**Table S1:** Overview of the linear mixed‐effects models used in the study. For each response variable, the table lists the fixed and random effects included in the fitted model structure. ‘Disturbance history’ refers to the control, long recovery and short recovery treatments. ‘Major pulse’ refers to the salinity pulse disturbance applied on Day 27 of the experiment. Time was included as either sampling day or centred sampling day (‘Day c’), depending on the model. For pairwise Aitchison distance and NST models, the random effect (1|comp) accounts for non‐independence among replicate pairs within each treatment × time combination. Here, comp identifies each unique replicate pair used to calculate pairwise community distances (e.g., Replicate 1–Replicate 2, Replicate 1–Replicate 3, Replicate 2–Replicate 3). For ASV richness models, the random effect (1|Replicate) accounts for repeated sampling of the same mesocosm over time.
**Table S2:** Results of permutational multivariate analysis of variance (PERMANOVA) on Aitchison distances testing the effects of disturbance history, major pulse treatment, time and their interactions across all sampling periods, before the major pulse (Days 1–27), and after the major pulse (Days 28–50).
**Table S3:** Results of permutational tests for homogeneity of multivariate dispersion (PERMDISP) based on Aitchison distances, testing differences in dispersion associated with disturbance history, major‐pulse treatment, and time across all sampling periods, before the major pulse (Days 1–27), and after the major pulse (Days 28–50).

## Data Availability

The data that support the findings of this study are openly available in National Center for Biotechnology Information (NCBI) at https://www.ncbi.nlm.nih.gov, reference number PRJNA531504.
