# Supplementary material for: Disturbance History Shapes Microbial Community Assembly and Predictability in a Continuous Culture System
Source: Environ Microbiol. 2026 Sep 27;28(10):e70430. doi: 10.1111/1462-2920.70430 (PMC13616742; doi:10.1111/1462-2920.70430)
Supplement: Supplementary file 1 [file EMI-28-e70430-s001.doc]

Supplementary Information

**Disturbance history shapes microbial community assembly and predictability in a continuous culture system**

Sophia Renes, Berenike Bick, Brendan McKie, Hannah B. Fried-Petersen, Silke Langenheder, David G. Angeler, Eva S. Lindström

Supplementary Methods S1: Library preparation

The first PCR step was performed using Illumina adapter attached bacterial primers 341F (Illumina adapter-N4-341F: 5’-ACACTCTTTCCCTACACGACGCTCTTCCGATCTNNNN CCTACGGGNGGCWGCAG-3’, following Herlemann et al. 2011 and 805NR (Illumina adapter-805NR: 5’-AGACGTGTGCTCTTCCGATCTGACTACNVGGGTATCTAATCC-3’, based on Apprill et al. 2015. These adapters target the V3 and V4 regions of the 16S ribosomal gene. Reactions were performed in triplicate in 20 µL volumes, using 1 x Q5® reaction buffer (New England Biolabs), 0.25 µM of each primer, 200 µM dNTPs (Thermo Fisher), 0.02 U/µL Q5® High-Fidelity DNA polymerase (New England Biolabs) and 1 µL sample. Initial denaturation took place at 98°C for 3 min, followed by 20 cycles of denaturation at 98°C for 10 s, annealing at 48°C for 30 s and extension at 72°C for 30 s. The final extension took place at 72°C for 2 min. After PCR step 1, pooled replicates were purified using magnetic beads. The second PCR step was performed to attach illumina handles and index primers to the purified samples from PCR 1 according to Sinclair et al. 2015 (Multiplex_fwd: AATGATACGGCGACCACCGAGA {TCTACAC}-[i5 index]ACACTC TTTCCCTACACGACG; Multiplex_rev: CAAGCAGAAG ACGGCATACGAGAT-[i7 index]-GTGACTGGAGTTCAGACGTGTGCTCTTCCGATCT). The second PCR step was also performed in triplicate in 20 µL volumes, using 1 x Q5 reaction buffer, 0.25 µM each of the forward and reverse index primers, 200 µM dNTPs, 0.02 U/µL DNA polymerase and 2 µL template DNA from PCR step 1. The second PCR program was as follows: 30 s initial denaturation at 98°C followed by 15 cycles of denaturation at 98°C for 10 s, annealing at 66°C for 30 s and extension at 72°C for 30 s. Final extension took place at 72°C for 2 min. Replicate PCR products from step 2 were pooled and purified using magnetic bead purification and quantified using Quant-iT™ PicoGreen® dsDNAReagent Kit (Invitrogen). The samples were divided into two pools for sequencing, where half of each treatment group per timepoint was randomly assigned to each pool to avoid possible systematic differences in the data caused by the different sequencing runs. After pooling samples at equimolar concentrations, the DNA was purified using gel purification before sending it for Illumina Miseq sequencing.

Supplementary Figures

*Figure S1.* Rarefaction curves of all samples (n=601), the red line shows the read number to which the samples were rarefied (10,245 reads).

*Figure S2.* ASV richness over time for the different disturbance-history treatments (grey = control, blue = long recovery, red = short recovery). Squares indicate samples collected before the major pulse on day 27. Following the major pulse, filled circles represent major-pulse treatments and open circles represent no-major-pulse treatments. Blue and red shaded areas indicate the timing of the minor salinity pulses in the long and short recovery treatments, respectively.

*Figure S3*. Relative abundances at phylum level in each replicate microcosm on the last day of the experiment (day 50) represented with bars, top plots who no disturbance history, middle long and bottom short recovery history, no major pulse treatments are presented on the left and major pulse treatments on the right side, colours indicate different phyla.

Figure S4. PCoA for a) before and b) & c) after the major pulse. The colour gradient indicates the days in the experiment and the symbols in a) and b) the different treatments (control, short and long recovery history) and in c) if the treatments did or did not receive the major pulse.

Supplementary Tables

*Table S1*. Overview of the linear mixed-effects models used in the study. For each response variable, the table lists the fixed and random effects included in the fitted model structure. “Disturbance history” refers to the control, long recovery, and short recovery treatments. “Major pulse” refers to the salinity pulse disturbance applied on day 27 of the experiment. Time was included as either sampling day or centred sampling day (“day c”), depending on the model. For pairwise Aitchison distance and NST models, the random effect (1|comp) accounts for non-independence among replicate pairs within each treatment × time combination. Here, comp identifies each unique replicate pair used to calculate pairwise community distances (e.g. Replicate1–Replicate2, Replicate1–Replicate3, Replicate2–Replicate3). For ASV richness models, the random effect (1|Replicate) accounts for repeated sampling of the same mesocosm over time.

*Table S2:* Results of permutational multivariate analysis of variance (PERMANOVA) on Aitchison distances testing the effects of disturbance history, major pulse treatment, time, and their interactions across all sampling periods, before the major pulse (days 1–27), and after the major pulse (days 28–50).

| **Effect** | **Df** | **Sum of squares** | **R2** | **F-value** | **p-value** |
| --- | --- | --- | --- | --- | --- |
| **All time points (day 1:50)** | | | | | |
| Disturbance history | 2 | 41406 | 0.04 | 15.01 | **0.001** |
| Major pulse | 1 | 23058 | 0.02 | 16.72 | **0.001** |
| Time | 1 | 82534 | 0.08 | 59.84 | **0.001** |
| Disturbance history x Major pulse | 2 | 32867 | 0.03 | 11.92 | **0.001** |
| Disturbance history × Time | 2 | 9527 | 0.01 | 3.45 | **0.001** |
| Major pulse × Time | 1 | 6322 | 0.01 | 4.58 | **0.001** |
| Disturbance history × Major pulse x Time |  |  |  |  |  |
| **Before pulse (day 1:27)** | | | | | |
| Disturbance history | 2 | 26793 | 0.05424 | 102.817 | **0.001** |
| Time | 1 | 48715 | 0.09862 | 373.887 | **0.001** |
| Disturbance history × Time | 2 | 9361 | 0.01895 | 35.924 | **0.001** |
| **After pulse (day 28:50)** | | | | | |
| Disturbance history | 2 | 24472 | 0.05247 | 86.659 | **0.001** |
| Major pulse | 1 | 20230 | 0.04337 | 143.273 | **0.001** |
| Time | 1 | 6534 | 0.01401 | 46.277 | **0.001** |
| Disturbance history x Major pulse | 2 | 24040 | 0.05154 | 85.128 | **0.001** |
| Disturbance history × Time | 2 | 4224 | 0.00906 | 14.958 | **0.01** |
| Major pulse × Time | 1 | 3852 | 0.00826 | 27.281 | **0.001** |
| Disturbance history × Major pulse x Time | 2 | 3252 | 0.00697 | 11.516 | 0.159 |

Table S3. Results of permutational tests for homogeneity of multivariate dispersion (PERMDISP) based on Aitchison distances, testing differences in dispersion associated with disturbance history, major-pulse treatment, and time across all sampling periods, before the major pulse (days 1–27), and after the major pulse (days 28–50).

| **Effect** | **Df** | **Residual Df** | **F-value** | **p-value** |
| --- | --- | --- | --- | --- |
| **All time points (day 1:50)** | | | | |
| Disturbance history | 2 | 598 | 5,419 | **0.006** |
| Major pulse | 1 | 599 | 8,443 | **0.009** |
| Time | 25 | 575 | 25,388 | **0.001** |
| **Before pulse (day 1:27)** | | | | |
| Disturbance history | 2 | 317 | 2,801 | 0.062 |
| Time | 13 | 306 | 54,134 | **0.001** |
| **After pulse (day 28:50)** | | | | |
| Disturbance history | 2 | 278 | 6,819 | **0.003** |
| Major pulse | 1 | 279 | 6,212 | **0.015** |
| Time | 11 | 269 | 0,366 | 0.962 |
